# History of one’s own performance modulates evaluative processing of another’s action outcomes, but not vice versa

**DOI:** 10.1038/s41598-021-03971-9

**Published:** 2022-01-07

**Authors:** Chikara Ishii, Jun’ichi Katayama

**Affiliations:** 1 Department of Psychological Science, Kwansei Gakuin University, Nishinomiya, 662-8501 Japan; 2 Center for Applied Psychological Science (CAPS), Kwansei Gakuin University, Nishinomiya, 662-8501 Japan

**Keywords:** Psychology, Neuroscience, Cognitive neuroscience, Social neuroscience

## Abstract

In action monitoring, i.e., evaluating an outcome of our behavior, a reward prediction error signal is calculated as the difference between actual and predicted outcomes and is used to adjust future behavior. Previous studies demonstrate that this signal, which is reflected by an event-related brain potential called feedback-related negativity (FRN), occurs in response to not only one's own outcomes, but also those of others. However, it is still unknown if predictions of different actors' performance interact with each other. Thus, we investigated how predictions from one’s own and another’s performance history affect each other by manipulating the task difficulty for participants themselves and their partners independently. Pairs of participants performed a time estimation task, randomly switching the roles of actor and observer from trial to trial. Results show that the history of the other’s performance did not modulate the amplitude of the FRN for the evaluation of one’s own outcomes. In contrast, the amplitude of the observer FRN for the other’s outcomes differed according to the frequency of one’s own action outcomes. In conclusion, the monitoring system tracks the histories of one’s own and observed outcomes separately and considers information related to one’s own action outcomes to be more important.

## Introduction

Rapid and accurate evaluation of the consequences of one’s own behavior and appropriate adjustment of subsequent behaviors are essential processes for human survival. Humans not only learn from their own mistakes but can also learn from others’ experiences^1,2^. Observational learning has been investigated not only in humans but also in other animals, and single-neuron recording in non-human primates has provided interesting findings regarding the representation of self and others in the brain^3–6^. For example, there are neurons that respond only to the errors of others in the medial frontal cortex^6^. Given that humans do not necessarily use only one source of learning when they adjust to an environment, it is important to clarify how they use information accumulated from multiple learning sources in an integrated manner. Examining such processes is crucial for understanding adaptive behaviors in a social environment. In the reinforcement learning theory of action monitoring^7^, prediction of an action’s outcome is the requisite element. In the present study, we investigated how predictions from one’s own and another’s performance history affect each other. To address this issue, we specifically evaluated (1) the effect of one’s own performance history on the evaluation of another’s action outcomes, and (2) the effect of another’s performance history on the evaluation of one’s own action outcomes using event-related brain potentials (ERPs).

Feedback-related negativity (FRN) is an electrophysiological signal used to study outcome evaluation processes. It is a negative deflection of the ERPs associated with negative outcomes such as monetary loss and performance error. It reaches maximum voltage at 200–300 ms after the presentation of an action’s outcome and has a front-central scalp distribution^8–10^. Normally, FRN amplitude is evaluated via the difference waveform created by subtracting the ERP for a positive outcome from the ERP for a negative outcome^11,12^. In addition, studies have reported that the source of this ERP is the anterior cingulate cortex (ACC)^8,9,13^. It should be noted that in this regard, several studies suggest that the difference in ERP amplitudes for desirable and undesirable outcomes is not due to FRN for the undesirable outcomes, but to reward positivity for desirable outcomes that correspond to activation of reward-related areas^14–18^. The reinforcement learning theory of action monitoring states that the FRN reflects a reward prediction error (RPE) signal—the difference between a predicted and an actual outcome. According to this theory, unexpected negative outcomes lead to larger FRN than expected negative outcomes. A study manipulated the outcome frequency with task difficulty to confirm the validity of this theory^19^. When a task is easy, participants are successful in many trials and should expect to be correct. In line with the theory, unpredicted erroneous feedback in the easy condition elicited larger amplitudes of FRN than that in the hard condition, followed by greater behavioral adjustments. Furthermore, recent studies using a modeling approach and single-trial analysis also have demonstrated that the FRN reflects the prediction error signals^20,21^. Hence, the perceived likelihood of an outcome is a determining factor for FRN amplitude.

A series of studies have revealed that the mechanism for the RPE calculation based on one’s own action outcomes also occurs when perceiving outcomes for others, resulting in a negative deflection for negative outcomes^22–24^. This negative deflection is called observer FRN (oFRN) since the latency, scalp distribution, and source are similar to the FRN^25^. Typically, the amplitude of the oFRN is much smaller than that of the FRN^22–24,26^. Findings on whether the oFRN is sufficiently sensitive to the expectancy of action outcomes are mixed as of the moment, with at least one study showing that the oFRN is less sensitive to the expectancy of action outcomes than the FRN^27^. However, another study demonstrated that unexpected outcomes elicited larger oFRN^28^. The effect of outcome expectedness on oFRN amplitude suggests that the system for generating the oFRN also stores the history of others’ performances and makes predictions regarding others’ action outcomes. However, it remains unclear whether the prediction of an action outcome distinguishes between different sources of experience or not.

In the present study, we examined the effect of the history of another’s performance on the RPE calculation derived from one’s own action outcomes by evaluating the FRN, and we evaluated the effect of one’s own performance history on the RPE derived from another’s action outcomes by evaluating the oFRN. We manipulated the task difficulty for pairs of participants independently. One participant always performed a time estimation task with either easy or hard difficulty. The other participant performed the same task with medium difficulty, making correct and erroneous responses equiprobably. If the prediction of action outcomes does not distinguish between sources, we would expect to see the same frequency effect in both the FRN and the oFRN, because the easy/hard difficulty of the one participant skews the overall distribution of correct and erroneous responses. That is, the history of one’s own and another’s performance would affect each other’s outcome predictions. In contrast, if the prediction of action outcomes does distinguish between sources, we would see no frequency effect or a different frequency effect between the FRN and the oFRN. We did not have a prediction as to which case would be upheld. We also explored how one’s own performance history would affect prediction of another’s outcomes, and how another’s performance history would affect the prediction of one’s own outcomes, if the latter case occurred.

## Methods

### Participants

Twelve gender-matched pairs (14 females and 10 males) participated in this experiment. Participants in each pair were independently recruited. Participants were 18–25 years old (*M* = 20.8; *SD* = 2.0) and reported having normal or corrected-to-normal vision. Prior to the experiment, we obtained written informed consent from participants. This experiment was approved by the University Research Ethics Review Board and was conducted in accordance with the Declaration of Helsinki. The data from one participant were not used in the analysis because the number of available trials for the computation of the ERPs did not reach the criteria.

### Procedure

**Figure 1:**
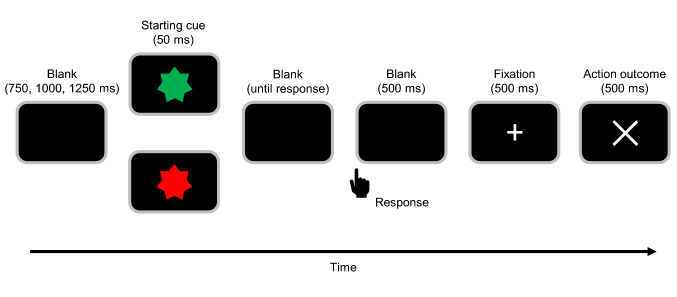
Timeline within a single trial in the time estimation task. Participants were asked to press a button 1 s after the presentation of a starting cue. A pair of participants looked at a red or green starting cue. The color of the cue determined which participant should respond in a trial.

Two participants sat side by side in front of a screen and performed a modified version of the time estimation task^19^. Electroencephalography (EEG) was recorded from both participants simultaneously. Figure 1 illustrates the sequence of events in a single trial of the time estimation task. In this task, participants must press a button after 1 s with a predefined error margin. Each trial began with a 50 ms starting cue followed by a blank screen until the participant responded. Prior to the experiment, one participant was assigned to a green button and the other to a red button. The starting cue for each trial was presented as a red or green visual stimulus and indicated which participant must perform the task. The color of the cue was randomly determined for each trial. Five hundred ms after the response, a fixation-cross was presented for 500 ms. Then, a visual feedback stimulus was presented for 500 ms. A circle for correct feedback was presented when the participant pressed the button within the predefined time window around 1 s, and a cross (X mark) for error feedback was presented when the response occurred outside the time window. Both participants would gain 8 JPY ($0.07) for each successful response. There was no monetary loss when a mistake occurred. Between trials a blank screen was presented for either 750, 1000, or 1250 ms, determined randomly.

In this experiment, we used three levels of task difficulty: easy, medium, and hard. Task difficulty determined whether the error margin within a response was close enough to 1 s to still be considered correct. This margin was adjusted accordingly if a response was correct or not. In the easy task, the error margin was set to 1000 ms ± 150 ms, and a correct response reduced this margin by 4 ms while an erroneous response increased it by 12 ms. In the medium task, the error margin started at 1000 ms ± 100 ms, and a correct/erroneous response reduced/increased the margin by 10 ms. In the hard task, the error margin started at 1000 ms ± 50 ms and decreased by 12 ms for a correct response and increased by 4 ms for an erroneous response.

The experiment started with a practice session of 20 trials with medium difficulty, followed by 720 trials divided over four regular sessions. Each session consisted of three blocks with 60 trials each. Task difficulty was varied over the four sessions. After each block, the cumulative amount of the accumulated reward would be displayed.

Task difficulty was varied across pairs in a specific manner. Each participant was subjected to two medium difficulty sessions and one easy and one hard session. The two participants were never assigned the same difficulty level concurrently; instead, one participant was always assigned to the medium difficulty session while the other always received the easy or hard session. Also, the two medium difficulty sessions always occurred consecutively, and either marked the first or last two regular sessions for a participant. The order of easy and hard sessions was counterbalanced between participants.

### Recording and analysis

Stimulus presentation and behavioral data collection were controlled by E-prime 2.0 (Psychology Software Tools, Inc., Pittsburgh, PA, USA). Mean response time and the number of correct responses were calculated for each participant and session. Trials with response times of less than 300 ms or greater than 3000 ms were excluded.

EEG was recorded from 32 Ag/AgCl electrodes (Fp1, Fp2, F7, F3, Fz, F4, F8, FT7, FC3, FCz, FC4, FT8, T7, C3, Cz, C4, T8, TP7, CP3, CPz, CP4, TP8, P7, P3, Pz, P4, P8, O1, Oz, O2, A1, and A2) according to the extended 10–20 system referenced to the nose tip using the electrode cap (Easy-cap, Asian cut) and the BrainAmp amplifier (Brain Products, Inc., Munich, Germany). The ground electrode was placed on AFz. Impedances were kept below 5 kΩ. The bandpass filter was set at 0.01 to 100 Hz and data were digitized at 1000 Hz.

Data processing was performed with the EEGLAB^29^ and ERPLAB toolboxes^30^ for MATLAB. The data were filtered with a 20 Hz low-pass filter (24 dB/octave) and segmented into 1000 ms epochs ranging from − 200 to 800 ms relative to feedback onset. We conducted an independent component analysis and removed components related to eye movements by visual inspection. Then, epochs in which the EEG exceeded ± 100 μV were excluded. The remaining epochs were averaged separately for each participant, factor, outcome (correct/error), and electrode location.

**Figure 2:**
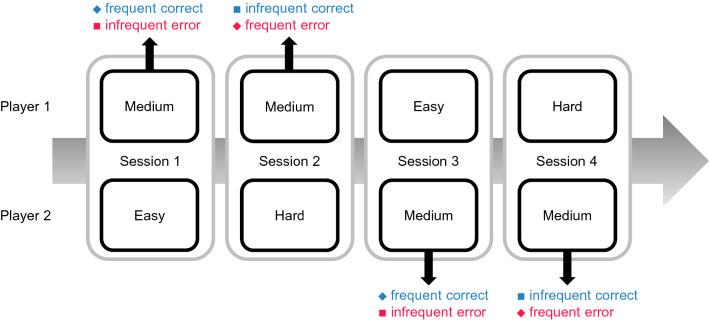
Example sequence of four sessions. One participant was always assigned medium difficulty while the other one was assigned easy or hard difficulty. In session 1, ERPs for one’s own frequent correct and infrequent error outcomes were extracted for Player 1 while ERPs for the partner’s frequent correct and infrequent error outcomes were extracted for Player 2. That is, the labels of frequency were determined by the bias of action outcomes in a session due to one participant performing the easy or hard difficulty.

We computed the difference waveforms by subtracting the correct- from the error-ERP over agency (self and other) and frequency (frequent and infrequent, see below). For the aim of current study, all difference waveforms were taken from the medium difficulty task (Fig. 2). In other words, ERPs for participants’ own outcomes were extracted from sessions in which they performed the medium difficulty task and the partner performed the easy and hard difficulty tasks. ERPs for their partners’ outcomes were extracted from sessions in which the partner performed the medium difficulty task and participants performed the easy and hard difficulty tasks. Then, the labels of frequency (frequent and infrequent) were defined by the bias in the overall outcome events due to one participant performing the easy or hard difficulty task. For example, when the task difficulty for the partner was easy, correct responses were frequent over the entire session while erroneous responses were infrequent. When the task difficulty for the partner was hard, correct responses were infrequent and erroneous responses were frequent. That means that the one’s own frequent ERP used the correct trials of the medium difficulty task when the partner was assigned the easy difficulty task and the error trials of the medium difficulty task when the partner was assigned the hard difficulty task. The distinction between self and other referred to who acted in the specific trial. All four difference waveforms were created according to this rule. ERPs from easy and hard difficulty tasks were not calculated because of the insufficient S/N ratio due to the small number of trials in either error or correct trials, respectively. To ensure an adequate signal to noise ratio, only participants who had at least 15 trials for each action outcome were used in the subsequent analysis, in line with previous studies^31,32^. According to this criterion, the data from one participant were excluded from further analysis.

First, we evaluated peak latencies of the difference FRN in the FCz with the jackknife procedure^33,34^. Twenty-three subground-averaged difference ERPs for a subsample of an *N* – 1 sample were computed. We defined the most negative peak between 150 and 300 ms as the peak latency of the difference FRN. Repeated measures ANOVA for the difference FRN peak latencies were conducted with Agency (self, other) × Frequency (frequent, infrequent). *F* values were adjusted in the ANOVA for peak latencies^34^. Because the peak latencies were explicitly different between agencies (see “Results”), we defined the difference FRN and oFRN amplitudes with different time windows. The amplitudes of the difference FRN (i.e., 224–274 ms) and the oFRN (i.e., 177–227 ms) were defined as the mean voltages in 50 ms time windows centered on the peak latencies on the grand average waveforms at the FCz site, respectively. Because of the large differences in the analyzed time windows, we conducted subsequent analyses separately for the FRN and the oFRN. First, we conducted one-sample *t* tests for both frequent and infrequent difference FRN amplitudes to confirm the significant FRN effect (i.e., difference between correct and erroneous responses). Then, we compared the frequent and infrequent difference FRN amplitudes with paired-sample *t* tests. In the same manner, we conducted the same statistical tests for the oFRN amplitudes as with the FRN amplitudes. The level of significance was set at 0.05. Partial eta squared (η_p_^2^) and Cohen’s *d* were reported as measures of effect size in the ANOVA and *t* tests, respectively.

## Results

### Behavioral data

The percentage of correct responses in the easy difficulty task (*M* = 74.4%, *SD* = 6.1) was much higher than in the hard difficulty task (*M* = 25.8%, *SD* = 1.7; *t*(22) = 39.2, *p* < 0.001, *d* = 10.913). There was no difference between the percentages of correct responses in the two medium difficulty tasks while the partner performed the easy (*M* = 50.6%, *SD* = 2.4) and hard (*M* = 50.8%, *SD* = 3.0) tasks (*t*(22) = 0.2, *p* = 0.801, *d* = 0.069). Participants earned 2,891 JPY ($26.5) on average. The response times for the easy (*M* = 1011, *SD* = 61) and hard (*M* = 990, *SD* = 117) tasks were not different (*t*(22) = 1.5, *p* = 0.158, *d* = 0.248). On the other hand, the response times while the partner performed the easy task (*M* = 995 ms, *SD* = 78) were shorter than the response times while the partner performed the hard task (*M* = 1030 ms, *SD* = 63; *t*(22) = 2.4, *p* = 0.027, *d* = 0.498).

### Electrophysiological data

**Figure 3:**
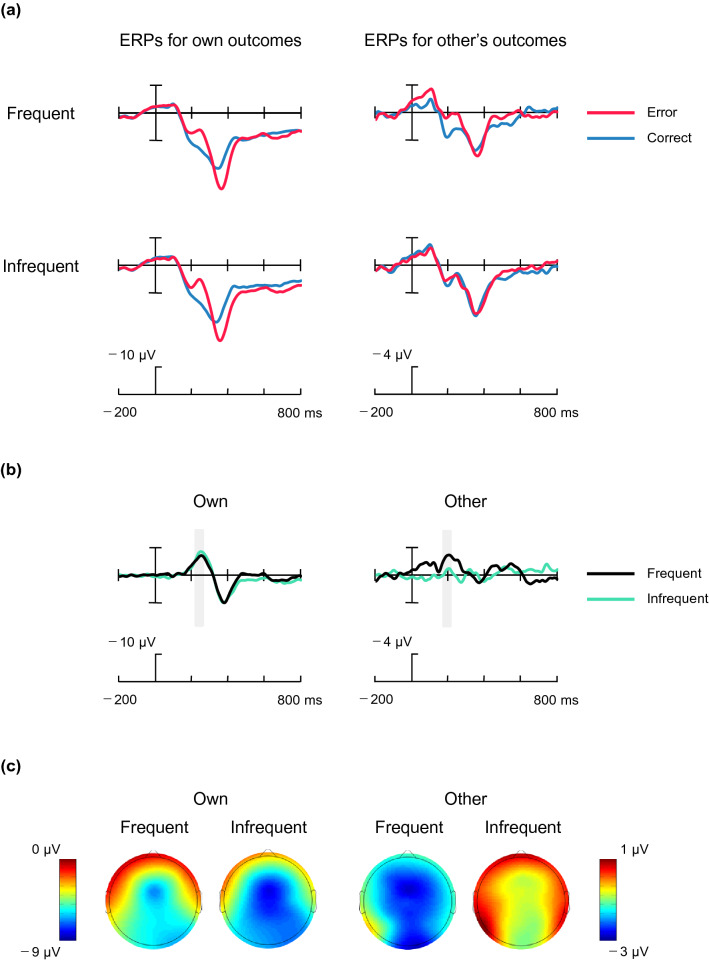
ERP waveforms and scalp distributions. (**a**) Grand-averaged ERPs elicited by correct and error feedbacks in the frequent and infrequent conditions at FCz. (**b**) Difference waveforms subtracting correct- from error-related ERPs for one’s own and the other’s outcomes. Gray areas indicate the time windows for statistical analyses. (**c**) Topographic maps indicate the scalp distributions of difference waveforms. The scaling is different for different actors.

Figure 3 shows the ERPs for one's own and another's correct and error feedbacks and their difference waveforms at FCz and their scalp distributions. Repeated measures ANOVA for the peak latency of the difference FRN showed a main effect of agency (*F*corrected(1,22) = 23.3, *p* < 0.001, η_p_^2^ = 0.514), indicating that the peak latency of the oFRN (*M* = 202 ms) was shorter than that of the FRN (*M* = 249 ms). The main effect of frequency and the interaction were not significant.

Significant elicitation of the FRN for both the frequent (− 6.72 μV) and infrequent (− 8.17 μV) conditions was confirmed by one-sample *t* tests (*t*(22) = 4.7, *p* < 0.001, *d* = 1.380; *t*(22) = 7.8, *p* < 0.001, *d* = 2.312). A paired-sample *t* test for the difference FRN amplitude for the frequent and infrequent conditions was not significant. Next, a one-sample *t* test confirmed the significant elicitation of the difference oFRN in the frequent (− 2.76 μV; *t*(22) = 3.3, *p* = 0.003, *d* = 0.986) but not in the infrequent condition (− 0.66 μV). The amplitude of difference oFRN in the frequent condition was significantly larger than that in the infrequent condition (*t*(22) = 2.4, *p* = 0.023, *d* = 0.575). To reveal the source of difference between the amplitude of difference oFRN in the frequent and infrequent conditions, we conducted an additional ANOVA for the original oFRN amplitude with Frequency and Outcome (correct, error) as factors. This test indicated significant main effect of outcome (*F*(1,22) = 7.3, *p* = 0.013, η_p_^2^ = 0.249) and interaction (*F*(1,22) = 6.0, *p* = 0.023, η_p_^2^ = 0.214). That is, the amplitude of oFRN for the frequent error outcome was larger than those for the frequent correct outcome (*p* = 0.003) and infrequent error (*p* = 024).

## Discussion

To investigate how the history of performance in oneself and others affects the RPE calculation derived from each other's action outcomes, we manipulated the task difficulty for self and other independently. The behavioral results showed that the task difficulty appropriately changed the frequency of the outcomes. The frequencies of correct and error trials were similar to those of a previous study^19^. Consistent with previous studies^8–12,19,22–24,26,27,31^, the typical FRN effect was found for one's own action outcomes, regardless of the partner’s outcome frequency. That is, the amplitude of the FRN for the error feedback was larger than that for the correct feedback, suggesting that feedback on one's own performance was processed appropriately. However, there was no difference between the difference FRN for the frequent and the infrequent conditions. On the other hand, the difference oFRN amplitude for the frequent condition was larger than that for the infrequent condition. If the responses and outcomes of self and other were tracked indistinguishably, then the same frequency effect should have emerged in the FRN and the oFRN. Thus, this effect in the difference oFRN amplitude, which is different from that in the FRN, suggests that the histories of own and another’s action outcomes are tracked separately.

We found a frequency effect in the amplitude of the difference oFRN. That is, the amplitude of the difference oFRN, derived from the other’s action outcomes, was larger in the frequent condition than in the infrequent condition. Moreover, the additional ANOVA for the original (i.e., pre-subtraction) oFRN amplitude indicated that the source of this frequency effect comes from differences in the evaluation of partner’s error outcomes rather than correct outcomes. A possible explanation of this phenomenon is that the monitoring system would predict the other’s action outcome relative to the history of one’s own performance. When a participant performed the hard difficulty task and had a negative-biased performance history, a moderate number of the other’s errors in the medium difficulty task were processed as a relatively unexpected event compared to one’s own erroneous responses. In the same manner, when a participant performed the easy difficulty task and had a positive-biased performance history, a moderate number of the other’s errors in the medium difficulty task were recognized subjectively as an expected event. Thus, the results of this study are consistent with previous studies indicating that unexpected outcomes lead to larger difference oFRN amplitudes than expected ones^28^. Several lines of research have suggested that the monitoring system uses a reference point to evaluate the consequence of an action^35,36^. For example, the absence of a reward is perceived as a bad event, or as a good event if the alternative is monetary loss^35^. Taken together, the present study suggests that the monitoring system calculating the RPE signal refers to the prediction for one’s own performance when it evaluates the other’s action outcomes.

Finally, one recent study with single trial EEG analysis indicates that the oFRN does not reflect the reward prediction errors^21^. However, in that study, the actor’s performance had no effect on the observer’s monetary gain or loss, and thus the other’s outcomes had only low significance. On the other hand, the reward prediction error may have been calculated for the partner’s outcomes as well since the partner’s outcomes also affected the monetary consequences for the observer in the current study. Apart from the findings from single trial EEG analysis, the sensitivity of the oFRN to the expectedness of outcomes was lower than that of the FRN^27^. Thus, the frequency effect of the oFRN, not the FRN, in this experiment may seem surprising at first glance. However, given that the person causing the bias in performance history in the session where we observed the frequency effect on the oFRN was oneself rather than the other, this result makes sense. These results suggest that information related to one’s own outcomes plays an important role even when predicting others’ outcomes. This implication extends the finding from previous studies that one’s own action outcomes are more motivationally significant than those of others^22–24,26^.

In conclusion, the present study revealed that the monitoring system tracked histories of one’s own and others’ outcomes separately. In addition, the information related to one’s own outcomes played a crucial role even when predicting the other’s action outcomes.

## Data Availability

The dataset generated and analyzed during the current study is accessible on the Open Science Framework (see http://osf.io/n2ebj).

## References

[1] Bandura A (1977). Social Learning Theory.

[2] Cross ES, Kraemer DJ, Hamilton AFDC, Kelley WM, Grafton ST (2009). Sensitivity of the action observation network to physical and observational learning. Cereb. Cortex..

[3] Chang SWC, Gariépy JF, Platt ML (2013). Neuronal reference frames for social decisions in primate frontal cortex. Nat. Neurosci..

[4] Ferrucci, L. *et al.* Dedicated representation of others in the macaque frontal cortex: From action monitoring and prediction to outcome evaluation. *Cereb. Cortex.* bhab253. 10.1093/cercor/bhab253 (2021). 10.1093/cercor/bhab253 PMC8841564 34428277

[5] Yoshida K, Saito N, Iriki A, Isoda M (2011). Representation of others’ action by neurons in monkey medial frontal cortex. Curr. Biol..

[6] Yoshida K, Saito N, Iriki A, Isoda M (2012). Social error monitoring in macaque frontal cortex. Nat. Neurosci..

[7] Holroyd CB, Coles MGH (2002). The neural basis of human error processing: Reinforcement learning, dopamine, and the error-related negativity. Psychol. Rev..

[8] Gehring WJ, Willoughby AR (2002). The medial frontal cortex and the rapid processing of monetary gains and losses. Science.

[9] Miltner WHR, Braun CH, Coles MGH (1997). Event-related brain potentials following incorrect feedback in a time-estimation task: Evidence for a “generic” neural system for error detection. J. Cogn. Neurosci..

[10] Hajcak G, Moser JS, Holroyd CB, Simons RF (2006). The feedback-related negativity reflects the binary evaluation of good versus bad outcomes. Biol. Psychol..

[11] Hajcak G, Holroyd CB, Moser JS, Simons RF (2005). Brain potentials associated with expected and unexpected good and bad outcomes. Psychophysiology.

[12] Yeung N, Holroyd CB, Cohen JD (2005). ERP correlates of feedback and reward processing in the presence and absence of response choice. Cereb. Cortex..

[13] Holroyd CB, Nieuwenhuis S, Yeung N, Nystrom L (2004). Dorsal anterior cingulate cortex shows fMRI response to internal and external error signals. Nat. Neurosci..

[14] Becker MPI, Nitsch AM, Miltner WHR, Straube T (2014). A single-trial estimation of the feedback-related negativity and its relation to BOLD responses in a time-estimation task. J. Neurosci..

[15] Holroyd CB, Pakzad-Vaezi KL, Krigolson OE (2008). The feedback correct-related positivity: Sensitivity of the event-related brain potential to unexpected positive feedback. Psychophysiology.

[16] Foti D, Winberg A, Dien J, Hajcak G (2011). Event-related potential activity in the basal ganglia differentiates rewards from nonrewards: Temporospatial principal components analysis and source localization of the feedback negativity. Hum. Brain Mapp..

[17] Carlson JM, Foti D, Mujica-Parodi LR, Harmon-Jones E, Hajcak G (2011). Ventral striatal and medial prefrontal BOLD activation is correlated with reward-related electrocortical activity: A combined ERP and fMRI study. Neuroimage.

[18] Proudfit GH (2015). The reward positivity: From basic research on reward to a biomarker for depression. Psychophysiology.

[19] Holroyd CB, Krigolson OE (2007). Reward prediction error signals associated with a modified time estimation task. Psychophysiology.

[20] Fischer AG, Ullsperger M (2013). Real and fictive outcomes are processed differently but converge on a common adaptive mechanism. Neuron.

[21] Burnside R, Fischer AG, Ullsperger M (2019). The feedback-related negativity indexes prediction error in active but not observational learning. Psychophysiology.

[22] Yu R, Zhou X (2006). Brain responses to outcomes of one’s own and other’s performance in a gambling task. NeuroReport.

[23] Fukushima H, Hiraki K (2009). Whose loss is it? Human electrophysiological correlates of non-self reward processing. Soc. Neurosci..

[24] Itagaki S, Katayama J (2008). Self-relevant criteria determine the evaluation of outcomes induced by others. NeuroReport.

[25] Koban L, Pourtois G (2014). Brain systems underlying the affective and social monitoring of actions: An integrative review. Neurosci. Biobehav. Rev..

[26] Leng Y, Zhou X (2010). Modulation of the brain activity in outcome evaluation by interpersonal relationship: An ERP study. Neuropsychologia.

[27] Bellebaum C, Colosio M (2014). From feedback-to response-based performance monitoring in active and observational learning. J. Cogn. Neurosci..

[28] Kobza S, Thoma P, Daum I, Bellebaum C (2011). The feedback-related negativity is modulated by feedback probability in observational learning. Behav. Brain. Res..

[29] Delorme A, Makeig S (2004). EEGLAB: An open source toolbox for analysis of single-trial EEG dynamics including independent component analysis. J. Neurosci. Methods..

[30] Lopez-Calderon J, Luck SJ (2014). ERPLAB: An open-source toolbox for the analysis of event-related potentials. Front. Hum. Neurosci..

[31] Ishii C, Katayama J (2020). Stimulus valence influences the evaluative processing of action outcome. NeuroReport.

[32] Marco-Pallares J, Cucurell D, Münte T, Strien N, Rodriguez-Fornells A (2011). On the number of trials needed for a stable feedback-related negativity. Psychophysiology.

[33] Miller JO, Patterson T, Ulrich R (1998). Jackknife-based method for measuring LRP onset latency differences. Psychophysiology.

[34] Ulrich R, Miller J (2001). Using the jackknife-based scoring method for measuring LRP onset effects in factorial designs. Psychophysiology.

[35] Holroyd CB, Larsen JT, Cohen JD (2004). Context dependence of the event-related brain potential associated with reward and punishment. Psychophysiology.

[36] Boksem MAS, Kostermans E, Cremer DD (2011). Failing where others have succeeded: Medial frontal negativity tracks failure in a social context. Psychophysiology.

